# Classification of crystal structure using a convolutional neural network

**DOI:** 10.1107/S205225251700714X

**Published:** 2017-06-13

**Authors:** Woon Bae Park, Jiyong Chung, Jaeyoung Jung, Keemin Sohn, Satendra Pal Singh, Myoungho Pyo, Namsoo Shin, Kee-Sun Sohn

**Affiliations:** a Faculty of Nanotechnology and Advanced Materials Engineering, Sejong University, Seoul 143-747, Republic of Korea; b Laboratory of Big-data Applications for Public Sector, Chung-Ang University, 221 Heukseok-dong, Dongjak-gu, Seoul 156-756, Republic of Korea; c Department of Printed Electronics Engineering, Sunchon National University, Chonnam 540-742 Republic of Korea; d Deep Solution Inc., 2636 Nambusunhwan-ro, Seocho-gu, Seoul 06738, Republic of Korea

**Keywords:** convolutional neural network (CNN), artificial neural network (ANN), powder X-ray diffraction, crystal system, inorganic materials, computational modelling, crystal structure prediction, properties of solids

## Abstract

A deep machine-learning technique based on a convolutional neural network (CNN) is introduced. It has been used for the classification of powder X-ray diffraction (XRD) patterns in terms of crystal system, extinction group and space group. About 150 000 powder XRD patterns were collected and used as input for the CNN with no handcrafted engineering involved, and thereby an appropriate CNN architecture was obtained that allowed determination of the crystal system, extinction group and space group. In sharp contrast with the traditional use of powder XRD pattern analysis, the CNN never treats powder XRD patterns as a deconvoluted and discrete peak position or as intensity data, but instead the XRD patterns are regarded as nothing but a pattern similar to a picture. The CNN interprets features that humans cannot recognize in a powder XRD pattern. As a result, accuracy levels of 81.14, 83.83 and 94.99% were achieved for the space-group, extinction-group and crystal-system classifications, respectively. The well trained CNN was then used for symmetry identification of unknown novel inorganic compounds.

## Abstract 2

A deep-machine-learning technique based on a convolutional neural network (CNN) is introduced. It has been employed for the classification of crystal system, extinction group and space group for given powder X-ray diffraction patterns of inorganic materials.

## 1. Introduction   

It would be a very difficult to describe an actual crystal structure perfectly using only powder X-ray diffraction (XRD) patterns as the raw data source, because the three-dimensional electron-density distribution is condensed into just one dimension in the powder diffraction pattern. Such data condensation leads to both accidental and exact peak overlap, which complicates the determination of individual peak intensities. This complication is the reason that the crystal symmetry (space group) cannot be obtained correctly from a powder XRD pattern for many low-symmetry phases, no matter what type of measurement tool is employed. Single-crystal diffraction data improve this complication and ease structural analysis compared with the use of powder diffraction. However, sample preparation for single crystals remains a challenge, although a small-sized single-crystal technique has recently become available for single-crystal XRD (Hirosaki *et al.*, 2014 ▸). It should also be noted that the most frequently encountered type of structural data in scientific and engineering fields is powder diffraction data, because the generally usable form of most engineering materials is in either a polycrystalline or a powder form.

A typical structural analysis for inorganic compounds should be able to extract the structural descriptors from the spectral descriptors. The typical structural descriptors are lattice parameters, overall symmetry and site symmetries, atomic type and position, site occupancy and thermal factor. Raw powder XRD pattern data are simplified by spectral descriptors such as peak position, height, width and shape, which are parameterized mathematically by introducing well defined peak profile functions such as pseudo-Voigt and others. The Rietveld refinement method (Rietveld, 1967 ▸, 1969 ▸) is known to treat powder XRD patterns not as discrete structure factors (*F_hkl_*) but as a full-profile continuous spectrum, and even includes some parameters designating the instrumental and sample conditions. Nonetheless, the Rietveld refinement method still employs only a number of discrete spectral descriptors, although the number of parameters is dramatically enhanced compared with other traditional analyses. More importantly, it should be noted that the utility of the Rietveld refinement method has been restricted to limited cases where the structure was roughly known. While we have successfully implemented the structure determination of many unknown novel compounds using either the direct method or the direct-space method (Park, Shin *et al.*, 2012 ▸; Park, Singh *et al.*, 2012 ▸; Park *et al.*, 2013 ▸, 2014 ▸), we find that initial steps such as indexing and space-group determination play crucial roles. These can be extremely difficult to establish, however, particularly in the presence of a small number of impurity phases with peaks that overlap the main phase.

There has been a great deal of progress in state-of-the-art software for indexing and space-group determination: *ITO* (Visser, 1969 ▸), *TREOR* (Werner *et al.*, 1985 ▸), *DICVOL* (Boultif & Louër, 1991 ▸), *McMaille* (Le Bail, 2004 ▸), *EXPO* (Altomare *et al.*, 2009 ▸) and *X-CELL* (Neumann, 2003 ▸). Despite this progress, correct indexing and the ensuing space-group determination require considerable expertise. In fact, the performance of the indexing software would be perfect under the premise that the correct choice of peaks has been secured. However, none of the auto-peak choice programs has provided us with satisfactory indexing results (please see the supporting information). Among all the above-mentioned indexing programs, *X-CELL* has been reported to be quite advantageous in terms of the consideration of impurity peaks, but this program also requires some sort of human intervention to provide impurity tolerance levels (*e.g.* 0–5 to reflect the number of allowed impurity peaks) during the indexing process for an acceptable outcome. This implies that human intervention is inevitable in judging peak positions and, more importantly, in sorting out peak overlap complications. There are other critical human intervention issues besides peak overlap complication, such as impurity peak identification, and these must be resolved in order to achieve a correct peak choice that will lead to reliable indexing and space-group determination. Without long-term experience, it would be nearly impossible to select only the correct peaks, which is particularly tricky for low-symmetry and large cell size materials with a certain amount of impurities.

In our opinion, the deep machine-learning technique could compensate for the incompleteness of rule-based powder XRD pattern interpretation. In this context, deep learning was introduced in the hope that it could outperform auto-peak-search-based indexing and the ensuing space-group determination without human intervention. The final goal of the present approach was to establish a deep-learning-based structure analysis platform, which would be easily accessible to non-experts who have only just begun to work in inorganic materials science, by providing them with an equal chance that only a well experienced expert might have grasped in the past.

Deep learning is a powerful set of techniques for learning in neural networks and it has proved to be a promising and effective tool that outperforms traditional rule-based methods in many areas, such as image classification, pattern recognition, speech recognition and natural language processing. Deep learning has recently become mainstream in the bio­logical and pharmaceutical research fields (Spencer *et al.*, 2015 ▸; Heffernan *et al.*, 2015 ▸; Mamoshina *et al.*, 2016 ▸). However, there have been no noticeable attempts to introduce deep-learning techniques into work on inorganic functional mat­erials. Deep learning is a form of modelling that is based on a convolutional neural network (CNN) (Lecun *et al.*, 1998 ▸). A CNN confers versatility for classification tasks and for discriminating among a number of classes (labels). In particular, a CNN works best for image classification and hand-written text identification. Weight sharing at certain layers of the network *via* filters (kernels) is key to a CNN, and this weight sharing makes it possible to build up a deep structure composed of many more layers than the conventional artificial neural network (ANN). Weight sharing through a kernel also allows a CNN to achieve an equivariance representation of basic feature data. In addition, either maximum- or average-pooling layers provide invariance to image (or pattern) transformations by reducing spatial resolution *via* down-sampling, details of which are given in the supporting information. CNNs have recently been successfully applied to large-scale image classification tasks (Krizhevsky *et al.*, 2012 ▸) and have yielded many state-of-the-art achievements in other areas. One of the most outstanding achievements has been AlphaGo (Silver *et al.*, 2016 ▸), which employed a CNN for both the policy and value networks in reinforcement learning and thereby defeated a human champion. Inspired by such successful achievements, we have developed an appropriate CNN to be used with powder XRD pattern classification, and have utilized it for crystal-system, extinction-group and space-group determination.

Prior to the boom in the art of deep learning, a number of powder XRD-related modelling studies used a conventional ANN. However, most of the previously reported cases were dealing with various feature engineering skills, such as manual featurization (Tatlier, 2011 ▸; Kustrin *et al.*, 2000 ▸), principal component analysis (PCA) (Obeidat *et al.*, 2011 ▸; Mitsui & Satoh, 1997 ▸; Chen *et al.*, 2005 ▸; Matos *et al.*, 2007 ▸), partial least-squares regression (PLSR) (Lee *et al.*, 2007 ▸) and various special statistical approaches (Gilmore *et al.*, 2004 ▸; Barr *et al.*, 2004 ▸). Feature engineering can simply be thought of as data contraction, which is more precisely defined as data-dimension contraction. It should also be noted that all of these previous machine-learning approaches were far removed from big-data analysis, and were restricted to a small data set consisting of manipulated data that shared common features, such as a small number of powder XRD patterns for mixtures consisting of a few previously well identified inorganic compounds. Less positively, all the previous machine learning for powder XRD pattern analyses has been associated with shallow ANNs. Consequently, the excessive feature engineering (dramatic data-dimension contraction), the shallow ANN and the small size of the training data set constituted a somewhat vicious circle, which imparted machine learning-based analysis with no merit by comparison with rule-based analysis prior to the advent of deep learning.

In contrast with such conventional approaches, we adopted a novel approach that coupled a deep convolutional neural network (CNN) with a seemingly overwhelming amount of powder XRD pattern data without the use of any handcrafted feature engineering. Neither data contraction nor knowledge-based data manipulation were involved in the preparation of the raw data for use in the CNN training. The full-profile powder XRD pattern was not treated as deconvoluted discrete peak-position and intensity data, but was instead regarded as nothing but a pattern, as if it were a picture. We prepared 150 000 powder XRD patterns that represented almost all of the inorganic compounds that exist on earth. Finally, we constructed a virtuous circle that was composed of no feature engineering (no data contraction), but only contained a deep CNN architecture, and big data. Such revolutionary and unprecedented CNN modelling for a powder XRD pattern classification enabled us to predict the crystal systems, the extinction groups and ultimately the space groups of totally unknown materials.

## 2. Data-set preparation   

To achieve a reliable CNN model, we prepared as much powder XRD pattern raw data as possible, with no feature engineering involved. The larger the data set, the more successful will be the modelling. It is unfortunate, however, that no database can provide raw data for powder XRD patterns. The International Centre for Diffraction Data (ICDD; http://www.icdd.com) does not allow subscribers to download all of their Powder Diffraction File (PDF) data in any type of primitive data file format. Therefore, we used the crystal structure solution data from the Inorganic Crystal Structure Database (ICSD; http://www.fiz-karlsruhe.de/icsd.html) to produce sufficient powder XRD pattern data for CNN modelling. In fact, the ICSD provided only structure solution data rather than experimental measured powder XRD patterns. It is practically impossible to collect an acceptable number of experimentally measured powder XRD patterns that would be sufficient for use in CNN learning. Thus the powder XRD patterns that we used for the CNN modelling were not experimentally measured real data, but were instead the calculated data from the structure solutions of every entry registered in the ICSD.

**Figure 1:**
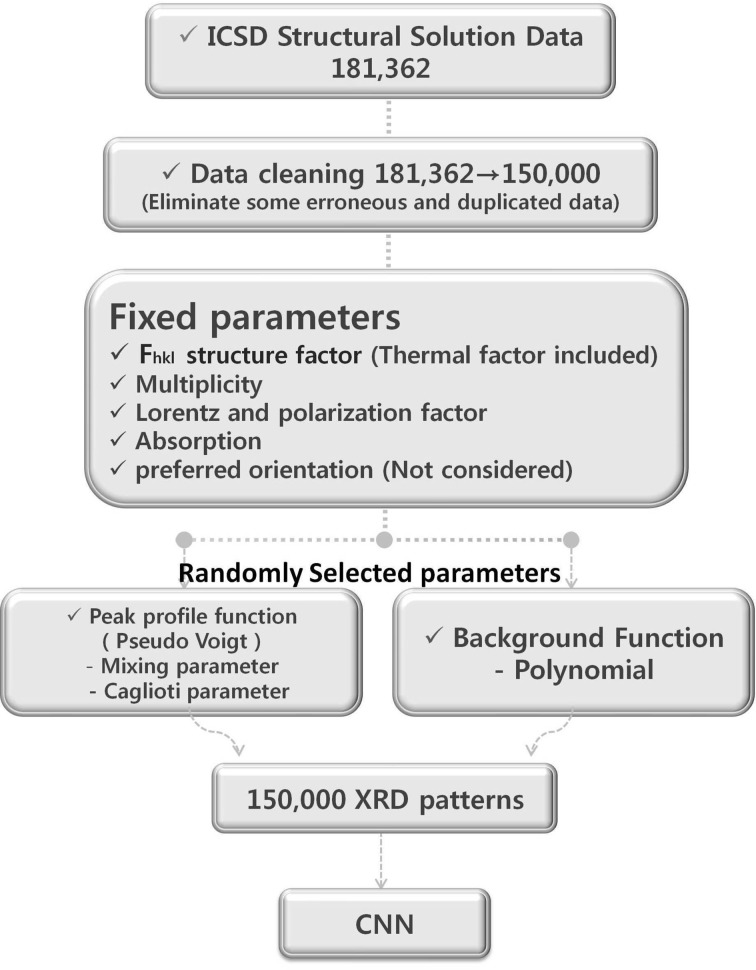
Schematic description of the acquisition of powder XRD data from the ICSD.

We produced a very large number of plausible powder XRD patterns calculated from the refined structure solution data. The structure solution data presented in the ICSD include symmetry information (space group), refined lattice parameters, atomic coordinates, occupancies and thermal factors. To simulate realistic powder XRD patterns from such a refined solution requires additional parameters such as the multiplicity for each peak, the Lorentz polarization factor, the preferred orientation, the background shape and the peak profile function. The first three parameters can be uniquely determined and fixed for each entry. The multiplicity can be obtained with ease from the symmetry data presented in the ICSD, the polarization correction was applied for laboratory XRD in the Bragg–Brantano geometry fitted with a graphite monochromator in the incident beam and the preferred orientation was considered to be non-existent. However, the background shape and the peak profile functions were varied randomly. The background was varied randomly using sixth-order polynomial functions. The peak profile function (pseudo-Voigt) was also varied by a random choice of mixing parameters as well as Caglioti parameters (Caglioti *et al.*, 1958 ▸). By adopting these random parameters, we produced ten slightly different powder XRD patterns for every single entry residing in the ICSD. Thereafter, we selected only one out of the ten and used it for CNN learning. Finally, Poissonian noise was added to the deterministic calculated pattern. Among the plausible powder XRD patterns created by all entries (181 362) registered in the ICSD up to January 2016, some erroneous and heavily duplicated data were eliminated. As a result, we finally secured 150 000 simulated powder XRD patterns. The entire procedure for the acquisition of these powder XRD data is described schematically in Fig. 1 ▸.

## 3. CNN architecture setup and test results for the powder XRD pattern classification   

The CNN for the powder XRD pattern classification is composed of an input layer, three pairs of convolutional and pooling layers, two fully connected layers, and an output layer. Each layer has a number of neurons that collect information from the previous layer. This information is converted into a specific value by using an activation function to be transferred to neurons in the next layer. The rectified linear unit (ReLu) has been a breakthrough in improving the performance of deep learning by replacing the conventional sigmoid activation function (Nair, 2010 ▸). This enhancement is doubled when a ReLu is coupled with a dropout that arbitrarily skips some neurons when conducting the back-propagation algorithm to derive the weight parameters of a CNN. The rate of the dropout was set at 30% in all three CNNs that were used for XRD classification.

**Figure 2:**
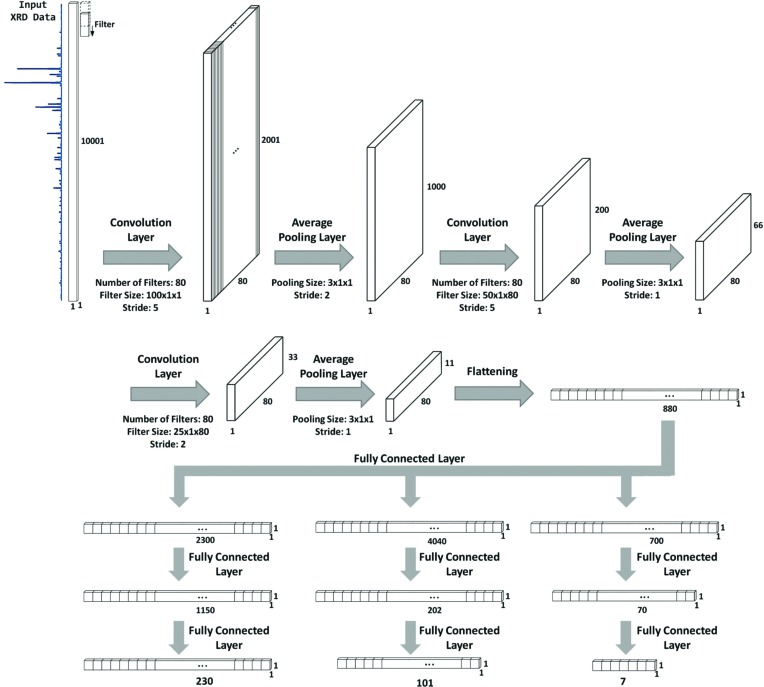
The CNN, composed of an input layer, three pairs of convolutional and pooling layers, two fully connected layers, and an output layer. Each layer has a number of neurons that collect information from the previous layer. This information is converted into a specific value using an activation function to be transferred to the neurons in the next layer. The filter size was halved from one layer to the next.

The performance of a CNN depends upon its architecture, which is based on the selection of hyper-parameters such as the numbers of convolutional, pooling and fully connected layers, the number of neurons in each layer, the size and number of convolutional filters with their stride size, and the rate of dropout. Unfortunately, there is no rigorous principle for determining the hyper-parameters. We chose them on a trial-and-error basis. The proposed architecture was determined after testing as many plausible versions as possible. The final versions of the architecture for the three different classifications are depicted in Fig. 2 ▸. Fortunately, the CNNs used for the three different structural classifications shown in Fig. 2 ▸ shared a common structure until flattening of the last pooling layer. They all had the same number (= 2) of fully connected layers, whereas the number of neurons in the layers differed. The output layer for each CNN was compatible with the number of classes (230 space groups, 101 extinction groups and seven crystal systems).

What follows is a description of how the proposed CNNs performed the XRD classification. Powder XRD data can be regarded as a string (vector), which is similar to a generic signal sequence. The dimensions of the XRD string are 10 001 × 1 × 1. The first convolution layer was created using eighty 100 × 1 × 1 filters, each of which slid through an input string with a stride value of 5. Each cell value of the convolutional hidden layer was computed by the linear combination of a filter’s weight and the values of the portion of a target sequence that the filter covered, and then the filter was activated by a ReLu. At this stage, each filter captured its own basic feature regardless of the feature location within a sequence. In addition, the adoption of filters had the advantage of reducing the number of weight parameters to be estimated, since each filter shared weight parameters wherever it resided (so-called weight-sharing). Following convolution, a new layer was created by pooling each of the 3 × 1 × 1 cells of the convoluted layer with average values, which had a smoothing effect on the original sequence and also provided a basis on which the subsequent convolutional filters could capture composite features by remote cells in the target sequence. The latter effect cannot be accommodated by mathematical tools based solely on a linear interaction between variables.

In the next stage, a second convolution layer was created by allowing eighty 50 × 1 × 80 filters to slide through the previous pooled layer. The second-level convolution filters extracted more complex features than those elicited from the first-level filters. The stride size (= 5) remained the same as the first convolutional layer. Following average pooling, a third convolution layer was created using eighty 25 × 1 × 80 filters. As shown in Fig. 2 ▸, the filter size was halved layer-by-layer. After average pooling again, the third convolutional hidden layer was flattened to facilitate connection to a fully connected hidden layer. The connection between the flattened layer and the next fully connected layer was the same as that between two consecutive hidden layers of a generic feed-forward neural network. After accommodating another fully connected layer, the second fully connected layer linearly fed neurons in the final output layer. The neurons of the final output layer were then activated with a soft-max function (Bridle, 1990 ▸), unlike the neurons within previous hidden layers which were activated by a ReLu. The final activation values from the soft-max function corresponded to the probability that an input data point belonged to each specific XRD class.

We tested the performance of the CNN model using a randomly selected test data set, the size of which amounted to 20% of the total data set. The test accuracies were evaluated to be 81.14, 83.83 and 94.99% for space-group, extinction-group and crystal-system classifications, respectively. This represents an amazingly accurate crystal-system prediction. However, the predictions for the extinction and space groups did not reach 90%. In fact, these numbers are very similar to the accuracy of human performance for the indexing and symmetry-determination process. It may appear that deep learning is little more than a mimicry of human behaviour, but it should be noted that, while what deep learning can do cannot surpass what humans can do, the efficiency and speed of a task are greatly improved when deep learning is substituted for learning by humans. However, it is certain that the CNN outperformed indexing based on auto-peak selection, as shown in sections S1 and S2 of the supporting information. Deep learning was prepared based on a personal coding using well established libraries, such as *Keras* and *tensorflow* in Python. The complete source code for our CNN model has been provided in the supporting information for convenience. Interested researchers may easily reproduce our CNN architecture using the above-mentioned libraries and test their own compounds for a tentative crystal system, extinction group and space group.

## 4. The structure-system, extinction-group and space-group classifications   

In principle, indexing is a process by which reflection indices, *hkl*, are assigned to all the peaks in a powder diffraction pattern. Accurate indexing leads to the correct determination of a crystal system and the correct estimation of lattice parameters. However, the lattice parameter was not taken as an output for this particular CNN, since our primary concern was a systematic classification of powder XRD patterns in terms of symmetry. In this regard, the activation function for the fully connected output layer in our CNN was the soft-max function, which represents probabilities rather than physical numbers. Therefore, precisely speaking, the CNN that we set up is not a model for indexing but is a classification platform to discern the crystal system, extinction group and space group of all the entries in the ICSD. In other words, the CNN could be a prediction model for the crystal systems, extinction groups and space groups of unknown inorganic compounds. This means that the labels for our XRD pattern data were comprised of the crystal system, the extinction group and the space group. The architecture of this CNN has three different output layers with seven, 101 and 230 neurons, which designate the probability density values for seven crystal systems, 101 extinction groups and 230 space groups, respectively. On the other hand, the input layer includes 10 001 neurons, which represent every intensity value in the 2θ range from 10 to 110°. The adoption of the full profile of the XRD pattern data as an input contrasts sharply with conventional analysis, which uses a dramatically contracted input vector *via* various feature engineering skills.

One might doubt why we employ a deep machine-learning technique for such a simple task as crystal-system determination, since the indexing can be completed perfectly well, using only a few peak positions, by the already well established commercial (or free) computational software packages, although determination of the ensuing extinction groups (or space groups) is difficult. Once ∼20–40 exact peak positions have been pinpointed, commercially available software enables us to index them with an acceptable figure of merit. The extinction group (or space group) can then be determined by checking the systematic absences. More precisely speaking, even when an exact extinction group can be identified, determination of the exact space group may be thwarted in many cases, because some extinction groups include a large number of probable space groups that are by definition not distinguishable.

Accurate peak selection is the most important prerequisite for reliable indexing and for determination of the exact extinction group (or space group). The correct peak selection for indexing could never be chosen without some type of human intervention (even sometimes expert intervention), because actual XRD pattern data always include distortions that cause uncertainties in judging peaks and pinpointing their exact positions. Examples include overlap (the most serious problem), a small number of impurity phases, data blurring due to the bad quality of measurement, and a researcher’s lack of crystallographic knowledge. Among all the indexing programs, *TREOR* requires much less time for indexing and is much more convenient for the initial level of indexing. The overall success rate of the *TREOR* program is better than 90%, and it is even higher than this for orthorhombic and higher symmetries (Werner *et al.*, 1985 ▸). The *TREOR* program, however, occasionally gives rise to unsatisfactory results in more complex systems (= lower-symmetry systems). Lower-symmetry systems usually result in many solutions with identical figures of merit and cell sizes. Thus, deciding the true solution can be very tricky, particularly for low-symmetry structures (please see the supporting information).

In order to show that conventional indexing software programs will fail without appropriate expert intervention, we employed two novel inorganic compounds that we had recently discovered, with exact crystal structures that had been clearly proven. These will be referred to as S-1 and S-2, Ca_1.5_Ba_0.5_Si_5_N_6_O_3_ (monoclinic *Cm*) (Park *et al.*, 2013 ▸) and Ba(Si,Al)_5_(O,N)_8_ (orthorhombic *A*2_1_ *am*) (Park *et al.*, 2014 ▸), respectively. The actual XRD patterns of these compounds even include some impurity peaks, although they are extremely small. We adopted an auto-peak-selection function to pinpoint the peak positions and used the *TREOR* program for indexing. We have tried indexing many times by varying the peak-selection conditions in the *TREOR* program, but have never had a correct indexing result. It is obvious that there is no way to index properly *via* auto-peak selection without human intervention. This failure could have originated from either the peak overlap or the impurity peaks. Of course, an expert in crystallography would never fail to index them correctly if many more trial-and-error efforts were made, as we did previously for these two compounds (Park *et al.*, 2013 ▸, 2014 ▸). Details of the indexing process on the synchrotron X-ray powder diffraction patterns of S-1 and S-2 are given in the supporting information.

As a matter of fact, the CNN model cannot perform the complete indexing of a powder XRD pattern, but simply identifies the crystal system, extinction group and space group. Both the choice of correct peaks and the ensuing consideration of systematic absences require a great deal of human expertise. However, our CNN model allows even novices to quickly reach the correct determination of a crystal system and probable space groups for novel compounds. It should be noted that our CNN is not intended to outperform experts who have much experience but, rather, it is meant to relieve their cumbersome burden in carrying out indexing and space-group determination. When non-experts are faced with unknown novel structures in the inorganic science research field, they can scarcely solve the structure given the current status of software development. Precisely speaking, our CNN model competes with neither well trained experts nor the well established indexing programs. The assistance is intended either for non-experts or for use with an auto-peak-search program. It is evident that, once a peak search has been correctly completed, then the *TREOR* program, or any of the other currently available indexing programs, would work perfectly.

Generally, it is difficult to account for why a CNN can classify a powder XRD pattern with a relatively high degree of accuracy. Deep-learning models have been criticized by many researchers as resembling a black box. The present study depended on deep CNN models and will not escape this criticism. The following explanation is intended to be a plausible answer to such criticism.

The CNN treats the powder XRD pattern as a sort of picture, rather than as a set of deconvoluted discrete peak position and intensity data, and thereby it excavates key features from the raw data through a number of filters. An implicit advantage of the use of CNN over conventional rule-based indexing comes from the fact that equal weight is placed not only on the low-angle data but also on the high-angle data, which are full of high-index peaks with tiny intensities and complicated multiple overlaps. In addition, the use of high-angle side data in the conventional rule-based indexing process creates some complications, which inherently originate from Bragg’s equation. For example, if we are working with a cubic material, then the following relationship holds
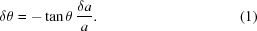
Based on this equation, a small erroneous change in the lattice parameter (*a*) can induce a huge change in the angle (θ). Therefore, the high-angle side data are considered to be problematic and difficult to treat. However, due to the pictorial consideration of the XRD patterns in the CNN, the higher-angle data are treated the same as all the other data.

A CNN is known to recognize features within an input sequence irrespective of scale and location. In particular, signals located far from each other in the original sequence could constitute a single hidden feature and a CNN could capture that feature. This can be recognized neither by human intuition nor by any shallow-learning model. Also, the filters of the convolution layers at higher angles were expected to capture features from small-scale signals in the input sequence, which has never been considered in rule-based XRD studies.

The filters were expected to abstract the features of an XRD profile, and the profile was then classified into a specific class *via* the last fully connected layer fed by the features. Each filter’s role of extracting a specific feature could be visualized by weight parameters. Each filter of a convolutional hidden layer had weights, each of which corresponded to a cell within the filter. For example, if the weight value of a filter cell is large, the filter captures a cell of the input sequence that is covered by the filter cell. On the other hand, if a filter cell value is small, the filter discards a cell of the input sequence that is covered by the filter cell.

**Figure 3:**
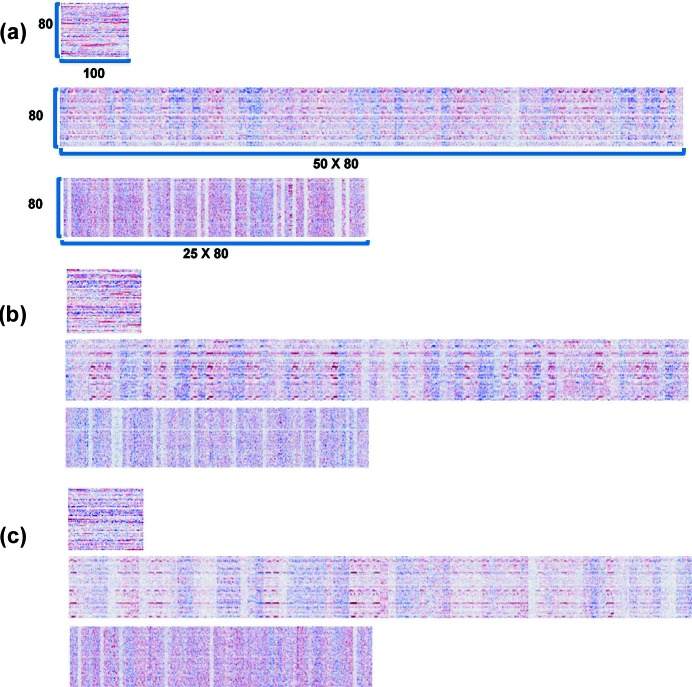
Filter visualizations of the three convolution layers. (*a*) The CNN for crystal-system classification, (*b*) for extinction-group classification and (*c*) for space-group classification. For each set of three, the top visualization shows 100 weights of 80 filters in the first convolution layer, the middle shows 50 × 80 weights of 80 filters in the second convolution layer and the bottom shows 25 × 80 weights of 80 filters in the third convolution layer. Red and blue represent different weights: a large positive value is represented by a strong red, whereas a large negative value is represented by a strong blue and white designates zero.

The abstracted features then contributed to classifying XRD patterns, although the features were not intuitively recognizable enough to be linked to the known characteristics of an XRD pattern. Fig. 3 ▸ depicts different features of the three convolution layers that were abstracted by each filter, where the weights are shown on a red-and-blue scale. A large positive value is represented by a strong red, whereas a large negative value is represented by a strong blue and white designates zero. The patterns at the top of Figs. 3 ▸(*a*), 3 ▸(*b*) and 3 ▸(*c*) depict 100 weights of 80 filters of the first convolution layer. Each row of the pattern represents a specific feature that the corresponding filter extracted from the input XRD profile sequence. The other elongated patterns show 50 × 80 and 25 × 80 weights of 80 filters of the second and the last convolution layers, respectively. Each row of these patterns indicates a more complex feature at a higher level than those extracted by a filter in the lower layers. Unfortunately, the visualized features look like random codes, but with a certain pattern that defies interpretation. These patterns might be successful in classifying the XRD pattern. It should be noted, however, that interpreting the performance of deep learning at the level of human intuition is meaningless.

Although we are not fully aware of how the CNN works, it is clear that it extracts information evenly from the full profile of data, which is in sharp contrast to the conventional indexing process where only a few low-angle peaks are taken into account. If a material of concern has a high symmetry with a relatively small unit cell then there would be fewer peaks available, which would make the indexing much easier for use of the conventional method. On the other hand, if the symmetry of an unknown material is low and the cell is large, then the number of peaks would be tremendously increased, and thereby the CNN would function as an auxiliary tool along with the conventional method.

## 5. Case study with actual XRD patterns of two novel structures   

**Figure 4:**
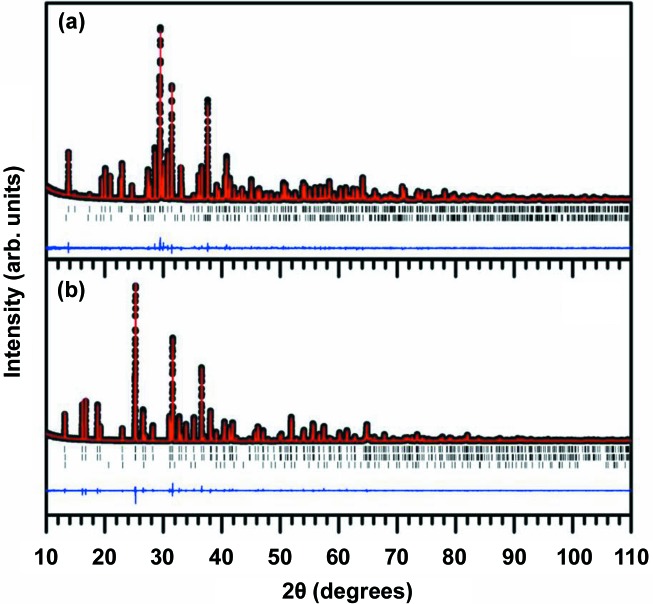
XRD patterns for Ca_1.5_Ba_0.5_Si_5_N_6_O_3_ (S-1) and BaAlSi_4_O_3_N_5_:Eu^2+^ system (S-2), along with the Rietveld refinement fits. The black dots, red lines, blue lines and vertical tick marks represent the experimental, calculated, difference profile and peak positions, respectively. The vertical tick marks in the second and third rows represent the peak positions corresponding to impurity phases.

Although we have already tested the performance of the trained CNN using a randomly selected test data set, an additional checkup is needed for a better understanding of our deep CNN model for prediction of the crystal system. For this double-check, we prepared two novel compounds (S-1 and S-2), which were discussed previously (Park *et al.*, 2013 ▸, 2014 ▸). The actual XRD patterns were measured experimentally for both S-1 and S-2 (Fig. 4 ▸) and were tested by the trained CNN. As a result, the CNN correctly predicted the crystal systems. It should be noted that we removed both S-1 and S-2 from the training data set and included them in neither the training nor the test data sets. Many entries in the ICSD exhibit similar compositions with similar structures. Such entries also give rise to similar powder diffraction patterns. In this regard, although test accuracies of 81.14, 83.83 and 94.99% were obtained for the space-group, extinction-group and crystal-system classifications, respectively, it was worthwhile to test whether or not our CNN could predict a completely unique structure with no similar structure types in the training set. However, it is very difficult to obtain experimental powder diffraction patterns of compounds that have unique structures. We pinpointed S-1 and S-2 because we had recently discovered them and been assured of their structural novelty and unique nature (Park *et al.*, 2013 ▸, 2014 ▸).

All entries in the ICSD are regularly categorized and those results are announced several times each year. Such a structural categorization principle is clearly based on reasonable crystallography-based principles (Allmann & Hinek, 2007 ▸). If an entry of concern belongs to one of the existing structural types (a so-called prototype), then the entry can be categorized into the prototype structure. The current number of prototype structures is 9093 according to the latest announcement in 2017 (ICSD web page). Most of the entries belong to one of these prototype structures, and a minor number of entries never belong to any of them. S-1 and S-2 belong to the latter case. This means that these two compounds have no similar compounds (XRD patterns) in the ICSD database and therefore had no similar patterns in our training data set. When the CNN made the correct determination of the structural system for these two, its applicability showed great promise. Although it is not clear whether such a wonderful result was fortuitous or not, the fact remains that our CNN gave us the correct crystal-system determination for these two.

The CNN architectures designed for the classification of extinction and space groups exhibited accuracies of 83.83 and 81.14%, respectively. However, both of these CNN architectures were unsuccessful in the confirmative test, and failed to predict the correct extinction and space groups for these two real XRD patterns. Nonetheless, we remain optimistic because a CNN with 101 or 230 neurons (nodes) at the output layer would require a deeper network architecture along with a much larger data set. We should be able to achieve success soon, in parallel with further advancements in computation capacity.

## 6. Conclusions   

In summary, three CNNs were developed for the space-group, extinction-group and crystal-system classification of 150 000 powder XRD patterns, and returned test accuracies of 81.14, 83.83 and 94.99%, respectively. The powder XRD patterns used for the CNNs were prepared using crystal structure data acquired from the ICSD, along with a set of random parameters for the background and peak width. In contrast with conventional structure analysis, the CNN-based space-group, extinction-group and crystal-system classification was accomplished by a totally data-based process from scratch. More importantly, it incorporated neither human (expert) interference nor assistance. Two actual powder XRD patterns of novel structures were tested, which belonged to none of the prototype structures listed in the ICSD and even involved a small amount of impurities. In these cases, the crystal-system prediction by the CNN was correct. The CNN read the entire raw powder XRD data as a picture, and it recognized the crystal system without the need for any theoretical analysis. This small success will be a milestone for further development of deep-learning-based analysis for many other conventional theoretical rule-based tasks in materials science.

## Supplementary Material

Additional figures and tables, and Python source code. DOI: 10.1107/S205225251700714X/fc5018sup1.pdf
