# Supplementary material for: Classification of crystal structure using a convolutional neural network
Source: IUCrJ. 2017 Jun 13;4(Pt 4):486–94. doi: 10.1107/S205225251700714X (PMC5571811; doi:10.1107/S205225251700714X)
Supplement: Supplementary file 1 [file m-04-00486-sup1.pdf]

IUCrJ

Volume 4 (2017)

Supporting information for article:

Classification of crystal structure using a convolutional neural network

Woon Bae Park, Jiyong Chung, Jaeyoung Jung, Keemin Sohn, Satendra Pal Singh, Myungho Pyo, Namsoo Shin and Kee-Sun Sohn

S1. Indexing of the synchrotron X-ray powder diffraction data on $Ca_{1.5}Ba_{0.5}Si_5N_6O_3$ system

The auto-peak-search was first carried out using proper peak profile parameters (peak threshold, background threshold and shoulder threshold) so as to exclude some of the weak intensity peaks of the $Ca_{1.5}Ba_{0.5}Si_5N_6O_3$ system as shown in Figure S1 (a). The peaks so obtained were then indexed using TREOR program. The indexing result ended up with no acceptable solution, but few probable solutions with very low figure of merits. The alternative choice of the profile parameter in the auto-peak-search procedure was then made to account for weaker intensity peaks as shown in Figure S1 (b) followed by indexing with TREOR program. The later choice also ended with no acceptable solution. However, the indexing result in the later case ended up with large number of probable solutions compared to former but having very low figure of merits. The output results obtained for the two case are shown in Table S1 and Table S2.The crystal structure of $Ca_{1.5}Ba_{0.5}Si_5N_6O_3$ system was later indexed with monoclinic crystal structure in the *Cm* space group with a great deal of human intervention.

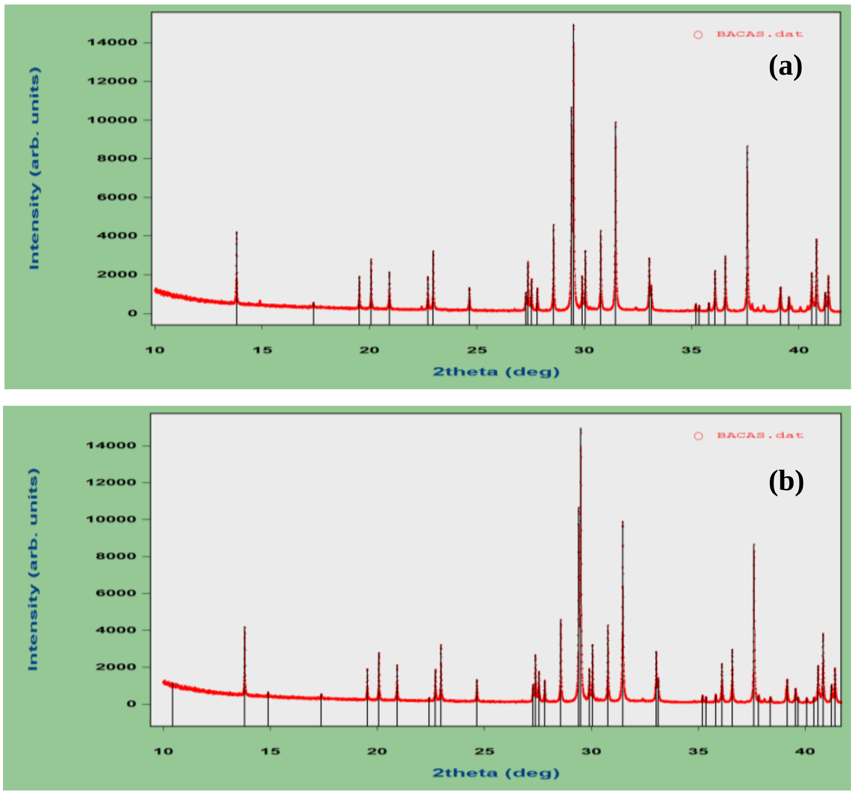

Figure S1 Peak positions of the XRD pattern obtained under different auto-peak-search parameters(peak threshold, background threshold and shoulder threshold) for the $Ca_{1.5}Ba_{0.5}Si_5N_6O_3$ system.

Table S1: Output of indexing results using peak positions of the $Ca_{1.5}Ba_{0.5}Si_5N_6O_3$ system shown in Figure S1 (a)

<table>
<tr><td rowspan="2">Peak Positions</td><td rowspan="2">Results</td><td colspan="6">Lattice parameters</td><td rowspan="2">Unit Cell Volume (Å^3)</td><td rowspan="2">M (20)</td><td rowspan="2">F (20)</td><td rowspan="2">Unindexed Peaks</td><td rowspan="2">Crystal System</td></tr>
<tr><td>a (Å)</td><td>b (Å)</td><td>c(Å)</td><td>α (°)</td><td>β (°)</td><td>γ (°)</td></tr>
<tr><td rowspan="3">13.807770
17.388229
19.530661
20.079321
20.923000
22.719839
22.974279
24.653040
27.280600
27.388439
27.556770
27.824011
28.579519
29.414089
29.512011
29.908590
30.056101
30.778820
31.469900
33.035568
33.126801
35.203629
35.364738
35.817169
36.102379
36.584320
37.608681
39.156448
39.547901
40.604530
40.830780
41.232979
41.383121</td><td>1</td><td>13.271821</td><td>11.932581</td><td>9.739318</td><td>90</td><td>90</td><td>90</td><td>5951.85</td><td>9</td><td>17</td><td>5</td><td>Orthorhombic</td></tr>
<tr><td>2</td><td>9.224786</td><td>23.865963</td><td>5.684216</td><td>90</td><td>98.038</td><td>90</td><td>1239.13</td><td>8</td><td>16</td><td>2</td><td>Monoclinic</td></tr>
<tr><td colspan="12">Comment: Some of the unindexed peaks in the above results do not belong to any impurity phase but the main phase, its negligence in the peak indexing should not be ignored. Thus the indexing results ended up with no acceptable solution. Please note unacceptably low figure of merit in the output results.</td></tr>
</table>

Table S2: Output of indexing results using peak positions of the $Ca_{1.5}Ba_{0.5}Si_5N_6O_3$ system shown in Figure S1 (b)

Peak Positions	Results	Lattice parameters						Unit Cell Volume (Å^3)	M (20)	F (20)	Unindexed Peaks	**Crystal System**
		a (Å)	b (Å)	c(Å)	α (°)	β (°)	γ (°)					
10.435100<br>13.807770<br>14.894670<br>17.388229<br>19.530661<br>20.079321<br>20.923000<br>22.426849<br>22.719839<br>22.974279<br>24.653040<br>27.280600<br>27.388439<br>27.556770<br>27.824011<br>28.579519<br>29.414089<br>29.512011<br>29.908590<br>30.056101<br>30.778820<br>31.469900<br>33.035568<br>33.126801<br>35.203629<br>35.364738<br>35.817169<br>36.102379<br>36.584320<br>37.608681<br>37.826149<br>38.377480<br>39.156448<br>39.547901<br>39.661140<br>40.074989<br>40.415131<br>40.604530<br>40.830780<br>41.232979<br>41.383121	1	16.880354	16.880354	20.887634	90	90	90	5951.85	9	19	4	**Tetragonal**
	2	15.286790	11.938805	9.134131	90	90	90	1667.03	7	14	5	**Orthorhombic**
	3	15.797421	11.932431	4.711424	90	104.566	90	859.57	17	33	6	**Monoclinic**
	4	15.807192	11.935061	9.430994	90	104.659	90	1721.33	7	14	2	**Monoclinic**
	5	13.837232	11.949532	6.787256	90	99.192	90	1107.85	7	15	5	**Monoclinic**
	6	13.492250	9.127168	12.479813	90	107.227	90	1467.89	6	13	0	**Monoclinic**
	7	9.200842	12.858434	8.940390	90	96.439	90	1051.05	6	12	8	**Monoclinic**

Comment: Some of the unindexed peaks in the above results do not belong to any impurity phase but the main phase, its negligence in the peak indexing should not be ignored. Thus the indexing results ended up with no acceptable solution. Please note unacceptably low figure of merit in the output results.

In conclusion, human intervention is initially required to identify each peak correctly (even the peaks with very weak intensities). After extensive analysis the final crystal structure was indexed with a monoclinic in the *Cm* space group having lattice parameters a = 7.07033(2) Å, b = 23.86709(7) Å, c = 4.825304(15) Å, α = γ = 90° and β = 109.0647(2)° and with figures of merit as **M(20) = 225 and F(20) = 432**.

S2. Indexing of the synchrotron X-ray powder diffraction data on $BaAlSi_4O_3N_5:Eu^{2+}$ system

Indexing of the peaks obtained through an auto-peak-search for the $BaAlSi_4O_3N_5:Eu^{2+}$ system shown in Figure S2 using the TREOR program ended up with a probable structural solution for the crystal system as monoclinic. In addition to this there were other solutions with identical figure of merit in the output file. All the solutions obtained in the output file of the TREOR program is shown in Table S3. The suggested solution (monoclinic crystal system) was not found to be valid since the observed and calculated data do not show good match in the structural refinement. The crystal structure of $BaAlSi_4O_3N_5:Eu^{2+}$ system was later indexed with orthorhombic system in the *A*21*am* space group, having the lattice parameters a = 9.48461(3) Å, b = 13.47194(6) Å, c = 5.77323(2) Å, and α = β = γ = 90° and figure of merit **M(20) = 110 and F(20) =179**. This brilliant solution was totally obtained by human intervention (i.e. our expertise), and the computational auto-peak-search can never reach this level.

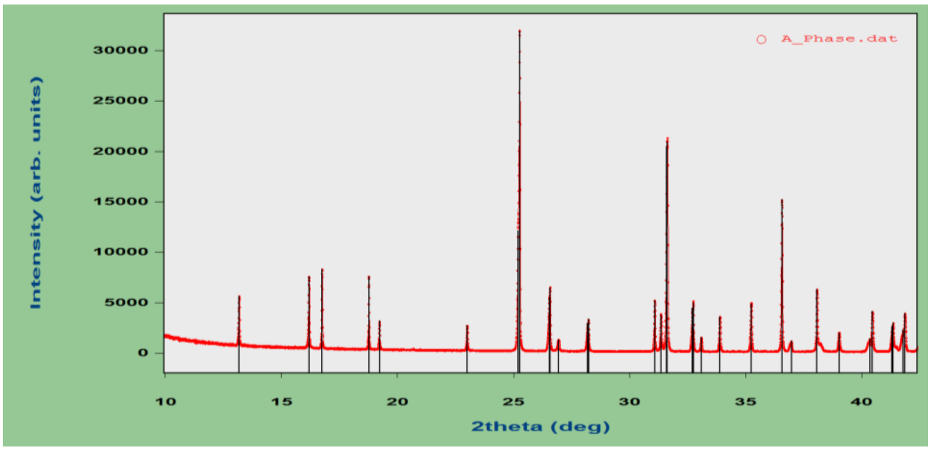

Figure S2 Peak positions of the XRD pattern obtained with an auto-peak-search for the $BaAlSi_4O_3N_5:Eu^{2+}$ system.

Table S3: Output of indexing results using peak positions of the $BaAlSi_4O_3N_5:Eu^{2+}$ system shown in Figure S2.

<table>
<tr><th rowspan="2">Peak Positions</th><th rowspan="2">Result</th><th colspan="6">Lattice parameters</th><th rowspan="2">Unit Cell Volume (Å^3)</th><th rowspan="2">M (20)</th><th rowspan="2">F (20)</th><th rowspan="2">Unindexed Peaks</th><th rowspan="2">Crystal System</th></tr>
<tr><th>a (Å)</th><th>b (Å)</th><th>c(Å)</th><th>α (°)</th><th>β (°)</th><th>γ (°)</th></tr>
<tr><td rowspan="6">13.188150
16.194180
16.761641
18.773069
19.228790
23.013981
25.197500
25.270580
26.545000
26.581079
26.946289
28.207331
28.237869
31.092190
31.363951
31.615000
31.639490
32.725319
32.755211
33.100208
33.908539
35.252048
36.572929
36.981800
38.084770
39.037060
39.046349
40.358879
40.469971
41.320000
41.368370
41.800320
41.874779</td><td>1</td><td>15.030773</td><td>15.030773</td><td>20.887634</td><td>90</td><td>90</td><td>90</td><td>2144.50</td><td>6</td><td>12</td><td>7</td><td>Tetragonal</td></tr>
<tr><td>2</td><td>9.496901</td><td>9.496901</td><td>19.174593</td><td>90</td><td>90</td><td>90</td><td>1729.38</td><td>7</td><td>14</td><td>6</td><td>Tetragonal</td></tr>
<tr><td>3</td><td>13.462188</td><td>9.488531</td><td>5.772748</td><td>90</td><td>90</td><td>90</td><td>737.39</td><td>20</td><td>39</td><td>4</td><td>Orthorhombic</td></tr>
<tr><td>4</td><td>13.474647</td><td>11.543999</td><td>9.488491</td><td>90</td><td>90</td><td>90</td><td>1475.95</td><td>16</td><td>32</td><td>5</td><td>Orthorhombic</td></tr>
<tr><td>5</td><td>7.174809</td><td>9.493328</td><td>6.809177</td><td>90</td><td>98.870</td><td>90</td><td>458.25</td><td>14</td><td>28</td><td>7</td><td>Monoclinic</td></tr>
<tr><td colspan="12">Comment: Among all the above five solutions given in the output file, the best solution for the crystal system was suggested as monoclinic (result no 5) by the TREOR program, even though it has 7 unindexed peaks. It should be noted that some of the unindexed peak in peak list do not belong to any impurity phase so its negligence in the peak indexing should not be ignored. The suggestion for the monoclinic system was perhaps based on the smallest cell volume obtained during indexing, in spite of the unacceptable figure of merit. However, further structural analysis using suggested parameters resulted in very bad match between the observed and experimental data, and it turned out to be an orthorhombic system.</td></tr>
</table>

S3. Basics of Artificial Neural Networks (ANNs) (Some of the text is taken from http://cs231n.github.io/)

Artificial neural network (ANN) is a machine learning approach that models human brain and consists of a number of artificial neurons (an information-processing unit that is fundamental to the operation of a neural network) that are linked together according to a specific network architecture. The objective of the neural network is to transform the inputs into meaningful outputs. ANNs are utilized in many scientific disciplines to solve a variety of problems such as pattern recognition, prediction, optimization etc.

Biological systems (humans/animals) are able to react adaptively to changes in their external and internal environment, and they use their nervous system to perform these behaviours. The basic computational unit of the brain is a **neuron (about 86 billion neurons in human nervous system)** are connected to synapses (10^{14} -10^{15}). Each neuron receives input signals from its **dendrites** and produces output signals along its single **axon.** The axon eventually branches out and connects via synapses to dendrites of other neurons. A schematic drawing of a biological neuron and a common mathematical model) is shown in Figure S3.

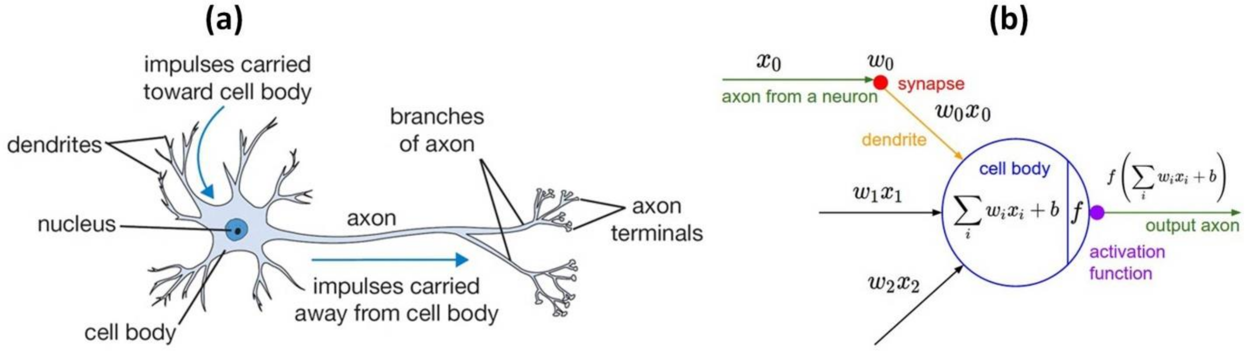

Figure S3 Schematic drawing of (a) a biological neuron and (b) a common mathematical model of neuron [Figures reproduced from http://cs231n.github.io/neural-networks-1/].

In the computational model of a neuron shown in Figure S3 (b), the neuron, input, output, and weight (w) are analogous to the cell body (also called soma), dendrite, axon and synapse respectively, to a biological system. The input signals that travel along the axons (e.g. x_0) interact multiplicatively (e.g. w_0x_0) with the dendrites of the other neuron based on the synaptic strength at that synapse (e.g. w_0). The idea is that the synaptic strengths (the weights w) are learnable and control the strength of influence (and its direction: excitory (positive weight) or inhibitory (negative weight)) of one neuron on another. In the basic model, the dendrites carry the signal to the cell body where they all get summed. If the final sum is above a certain threshold, the neuron can *fire*, sending a spike along its axon and frequency of the firing communicates information. Similarly, the neuron computes the weighted sum of the input signals and compares the result with a threshold value. If the net input is less than the threshold, the neuron output is –1. But if the net input is greater than or equal to the threshold, the neuron becomes activated and its output attains a value +1. Commonly used **activation functions** in neural networks are step function**,** Sigmoid function, $\sigma(x)=1/(1+e^{-x})$ (it takes a real-valued number and "squashes" it into range between 0 and 1), *tanh* (it squashes a real-valued number to the range [-1, 1]), etc. The Rectified Linear Unit (ReLu) has become very popular in the last few years, it computes the function $f(x)=max(0,x)$. In other words, the activation is simply thresholded at zero.

Neural Networks are modeled as collections of neurons that are connected in an acyclic graph. For regular neural networks, the most common layer type is the fully-connected layer in which neurons between two adjacent layers are fully pair wise connected, but neurons within a single layer share no connections. Figure S4 shows two example of Neural Network topologies that use a stack of fully-connected layers.

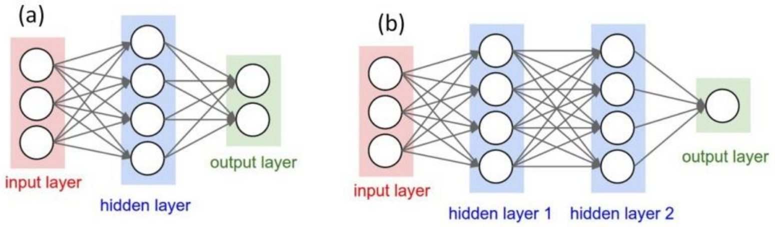

Figure S4 (a) A 2-layer Neural Network consisting of three inputs, one hidden layer of 4 neurons (or units) and one output layer with 2 neurons.
(b)A 3-layer neural network consisting of three inputs, two hidden layers of 4 neurons each and one output layer. [Figures reproduced from http://cs231n.github.io/neural-networks-1/]

S4. Detailed description of the Convolutional Neural Networks (CNNs) [Some of the text is taken from http://cs231n.github.io/)]

Convolutional Neural Networks are very similar to an ordinary Neural Networks described in the previous section: they are made up of neurons that have learnable weights and biases. Each neuron receives some inputs, performs a dot product and optionally follows it with a non-linearity. The whole network still expresses a single differentiable score function: from the raw image pixels on one end to class scores at the other. And they still have a loss function (e.g. Support vector matrix (SVM)/Softmax) on the last (fully-connected) layer and all the tips/tricks that were developed for learning regular Neural Networks still apply. The only difference is that the CNN architectures make the explicit assumption that the inputs are images, which allows to encode certain properties into the architecture. These then make the forward function more efficient to implement and vastly reduce the amount of parameters in the network.
As describes in the previous section that a Neural Networks receive an input (a single vector), and transform it through a series of hidden layers. Each hidden layer was made up of a set of neurons, where each neuron was fully connected to all neurons in the previous layer, and where neurons in a single layer function completely independently and do not share any connections. The last fully-connected layer is called the “output layer” and in classification settings it represents the class scores. *Regular Neural Nets could work well with images with small size*. For example, images with size 32x32x3 (32 wide, 32 high, 3 color channels), a single fully-connected neuron in a first hidden layer of a regular Neural Network would have 32*32*3 = 3072 weights and could be manageable. However, an image with large size for example 200x200x3, would lead to neurons that have 200*200*3 = 120,000 weights. We certainly want to have several such neurons, so the parameters would add up quickly. Clearly, this full connectivity is wasteful and the huge number of parameters would quickly lead to overfitting.

Convolutional Neural Networks on the other hand take advantage of the fact that the input consists of images and they constrain the architecture in a more sensible way. In particular, unlike a regular Neural Network, the layers of a CNN have neurons arranged in 3 dimensions: **width, height, depth** as shown in Figure S5. The word *depth* here refers to the third dimension of an activation volume, not to the depth of a full Neural Network, which can refer to the total number of layers in a network.

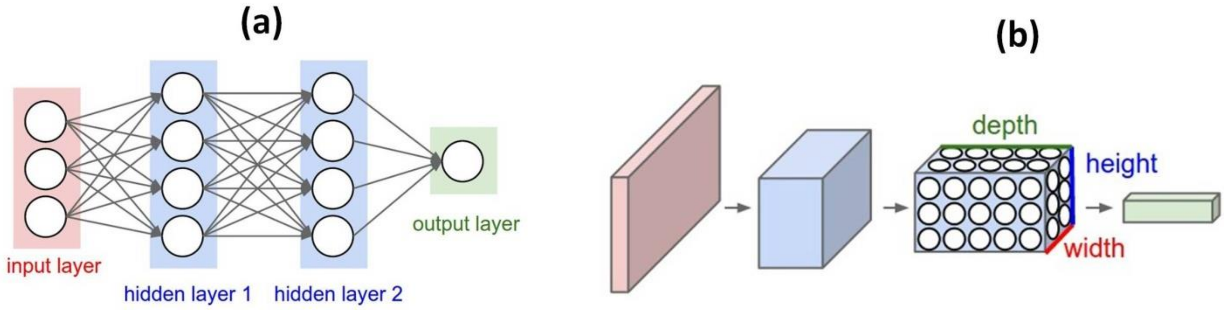

Figure S5(a) A regular 3-layer Neural Network, **(b)** A CNN arranges its neurons in three dimensions (width, height, depth), as visualized in one of the layers. Every layer of a CNN transforms the 3D input volume to a 3D output volume of neuron activations. In this example, the red input layer holds the image, so its width and height would be the dimensions of the image, and the depth would be 3 (Red, Green, Blue channels). [Figures reproduced from http://cs231n.github.io/convolutional-networks/]

A simple CNN is a sequence of layers, and every layer of a CNN transforms one volume of activations to another through a differentiable function. Basically three main types of layers are used to build CNN architectures: **Convolutional Layer**, **Pooling Layer**, and **Fully-Connected Layer** (exactly as seen in regular Neural Networks). All these layers are stacked to form a full CNN **architecture**. Example of CNN for classification of image of a car is shown Figure S6. CNNs transform the original image layer by layer from the original pixel values to the final class scores. The parameters in the CONV/FC layers will be trained with gradient descent so that the class scores that the CNN computes are consistent with the labels in the training set for each image.

Example:
A simple CNN for image classification could have the architecture [INPUT - CONV - RELU - POOL - FC].

- INPUT [e.g 32x32x3 image size] will hold the raw pixel values of the image, in this case an image of width 32, height 32, and with three color channels R,G,B.
- CONV layer will compute the output of neurons that are connected to local regions in the input, each computing a dot product between their weights and a small region they are connected to in the input volume. This may result in volume such as [32x32x12] if we decided to use 12 filters.
- RELU layer will apply an element wise activation function, such as the max(0,x) thresholding at zero. This leaves the size of the volume unchanged ([32x32x12]).

- POOL layer will perform a downsampling operation along the spatial dimensions (width, height), resulting in volume such as [16x16x12].

- FC (i.e. fully-connected) layer will compute the class scores, resulting in volume of size with class score.

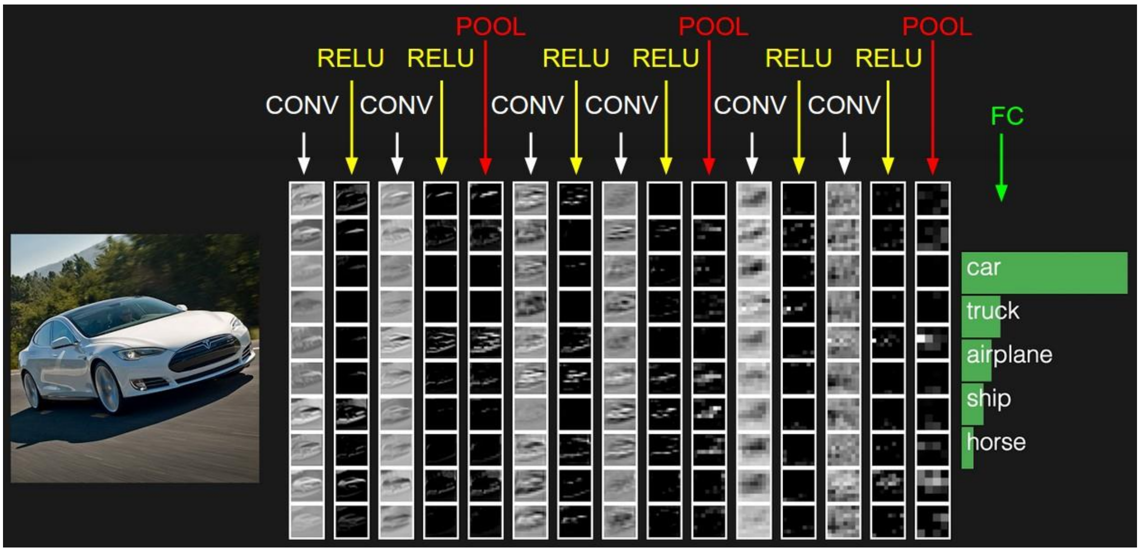

Figure S6 The initial volume stores the raw image pixels (left) and the last volume stores the class scores (right). Each volume of activations along the processing path is shown as a column. Since it's difficult to visualize 3D volumes, each volume's slices is laid out in rows. The last layer volume holds the scores for each class for the sorted top 5 scores [Figures reproduced from http://cs231n.github.io/convolutional-networks/].

S5. Training methods

Both supervised and supervised process may be adopted for the training. In the supervised training, both the inputs and the outputs are provided. The network then processes the inputs and compares its resulting outputs against the desired outputs. Errors (the difference of the desired output and the given output) are then propagated back through the system, causing the system to adjust the weights which control the network. This process occurs over and over as the weights are continually tweaked. The set of data which enables the training is called the training set. During the training of a network the same set of data is processed many times as the connection weights are ever refined. Whereas, in unsupervised training, the network is provided with inputs but not with desired outputs. The network is required to self-organise (i.e. to teach itself) depending on some structure in the input data.This is often referred to as self-organization or adaption.

S6. Python source code for the XRD classification

```
#import basic library
import numpy as np
import pandas as pd
from random import random
import bottleneck as bn
import heapq

#load y_label
docX = []
docY = []

df_out = pd.read_csv('D://JI//newxrd//150000Y.csv',header=None)
filename = df_out[0]
Y_data230 = np.array(df_out[1]) - 1
Y_data7 = np.array(df_out[2]) - 1
Y_data101 = np.array(df_out[3]) - 1

#load X_data
for i in filename :
    filepath = "D://JI//newxrd//fresh_random//" + str(i) + ".csv"
    arr = pd.read_csv(filepath,header=None)
    arr = np.array(arr)
docX.append(arr)
X_data = np.array(docX)
docX = []

#randomly choose 20% test data
tot_ix =range(len(Y_data7))
test_ix = np.random.choice(tot_ix, int(len(Y_data7)*0.2), replace=False)
test_ix = np.sort(test_ix,axis=0)
train_ix = list(set(tot_ix) - set(test_ix))

#write test data index into csv files
test_ix = np.reshape(test_ix, test_ix.shape + (1,))
mat1 = test_ix
dataframe1 = pd.DataFrame(data=mat1.astype(int))
dataframe1.to_csv('choose20percenttestset.csv', sep=',', header=False,
float_format = '%.2f', index = False)

#load test index and convert to hot vector
test_index = pd.read_csv('choose20percenttestset.csv', header=None)
test_ix = test_index[0]

tot_ix = range(len(Y_data7))
train_ix = list(set(tot_ix) - set(test_ix))
test_X = X_data[test_ix]
train_X = X_data[train_ix]

test_Y7 = Y_data7[test_ix]
train_Y7 = Y_data7[train_ix]
```

```
test_Y101 = Y_data101[test_ix]
train_Y101 = Y_data101[train_ix]

test_Y230 = Y_data230[test_ix]
train_Y230 = Y_data230[train_ix]

from keras.utils.np_utils import to_categorical

train_Y7 = to_categorical(train_Y7, 7)
test_Y7 = to_categorical(test_Y7, 7)

train_Y101 = to_categorical(train_Y101, 101)
test_Y101 = to_categorical(test_Y101, 101)

train_Y230 = to_categorical(train_Y230, 230)
test_Y230 = to_categorical(test_Y230, 230)

#shuffle data before training
tot_ix =range(len(train_X))
rand_ix = np.random.choice(tot_ix, len(train_X), replace=False)
train_X = train_X[rand_ix]
train_Y101 = train_Y101[rand_ix]
train_Y7 = train_Y7[rand_ix]
train_Y230 = train_Y230[rand_ix]

#import keras library
from keras.models import Sequential
from keras.layers import Input, Dense, Flatten, Merge, merge
from keras.layers import Dropout, Activation
from keras.layers import Convolution1D, MaxPooling1D, AveragePooling1D
from keras.layers import ZeroPadding1D
from keras.layers.noise import GaussianNoise
from keras.optimizers import SGD
import keras.callbacks
from keras.models import Model

# 7 label training
model = Sequential()

model.add(Convolution1D(80, 100, subsample_length = 5, border_mode =
'same', input_shape=(10001,1))) #add convolution layer
model.add(Activation('relu')) #activation
model.add(Dropout(0.3))
model.add(AveragePooling1D(pool_length=3, stride=2)) #pooling layer

model.add(Convolution1D(80, 50, subsample_length = 5, border_mode =
'same'))
model.add(Activation('relu'))
model.add(Dropout(0.3))
model.add(AveragePooling1D(pool_length=3, stride=None))

model.add(Convolution1D(80, 25, subsample_length = 2, border_mode =
'same'))
model.add(Activation('relu'))
```

```
model.add(Dropout(0.3))
model.add(AveragePooling1D(pool_length=3, stride=None))

model.add(Flatten())
model.add(Dense(700))
model.add(Activation('relu'))
model.add(Dropout(0.5))

model.add(Dense(70))
model.add(Activation('relu'))
model.add(Dropout(0.5))

model.add(Dense(7))
model.add(Activation('softmax'))


#Compile
model.compile(loss='categorical_crossentropy', optimizer='Adam',
metrics=['accuracy'])

#fit
filepath='D://JI//newxrd//xrd_model//7labelmodel.out'
modelCheckpoint=keras.callbacks.ModelCheckpoint(filepath,
monitor='val_loss', verbose=0, save_best_only=True, mode='auto')
history = keras.callbacks.History()
model.fit(train_X, train_Y7, batch_size=500, nb_epoch=5000,
validation_split=0.25, callbacks=[modelCheckpoint,history], shuffle=True)

# check the accuracy
a = model.evaluate(train_X, train_Y7)
print(a)
a = model.evaluate(test_X, test_Y7)
print(a)

# 101 label training
model = Sequential()

model.add(Convolution1D(80, 100, subsample_length = 5, border_mode =
'same', input_shape=(10001,1))) #add convolution layer
model.add(Activation('relu')) #activation
model.add(Dropout(0.3))
model.add(AveragePooling1D(pool_length=3, stride=2)) #pooling layer

model.add(Convolution1D(80, 50, subsample_length = 5, border_mode =
'same'))
model.add(Activation('relu'))
model.add(Dropout(0.3))
model.add(AveragePooling1D(pool_length=3, stride=None))

model.add(Convolution1D(80, 25, subsample_length = 2, border_mode =
'same'))
model.add(Activation('relu'))
model.add(Dropout(0.3))
model.add(AveragePooling1D(pool_length=3, stride=None))
```

```
model.add(Flatten())
model.add(Dense(4040))
model.add(Activation('relu'))
model.add(Dropout(0.5))

model.add(Dense(202))
model.add(Activation('relu'))
model.add(Dropout(0.5))

model.add(Dense(101))
model.add(Activation('softmax'))


#Compile
model.compile(loss='categorical_crossentropy', optimizer='Adam',
metrics=['accuracy'])

#fit
filepath='D://JI//newxrd//xrd_model//101labelmodel.out'
modelCheckpoint=keras.callbacks.ModelCheckpoint(filepath,
monitor='val_loss', verbose=0, save_best_only=True, mode='auto')
history = keras.callbacks.History()
model.fit(train_X, train_Y101, batch_size=800, nb_epoch=5000,
validation_split=0.25, callbacks=[modelCheckpoint,history], shuffle=True)

# check the accuracy
a = model.evaluate(train_X, train_Y101)
print(a)
a = model.evaluate(test_X, test_Y101)
print(a)


# 230 label training
model = Sequential()

model.add(Convolution1D(80, 100, subsample_length = 5, border_mode =
'same', input_shape=(10001,1))) #add convolution layer
model.add(Activation('relu')) #activation
model.add(Dropout(0.3))
model.add(AveragePooling1D(pool_length=3, stride=2)) #pooling layer

model.add(Convolution1D(80, 50, subsample_length = 5, border_mode =
'same'))
model.add(Activation('relu'))
model.add(Dropout(0.3))
model.add(AveragePooling1D(pool_length=3, stride=None))

model.add(Convolution1D(80, 25, subsample_length = 2, border_mode =
'same'))
model.add(Activation('relu'))
model.add(Dropout(0.3))
model.add(AveragePooling1D(pool_length=3, stride=None))
```

```
model.add(Flatten())
model.add(Dense(2300))
model.add(Activation('relu'))
model.add(Dropout(0.5))

model.add(Dense(1150))
model.add(Activation('relu'))
model.add(Dropout(0.5))

model.add(Dense(230))
model.add(Activation('softmax'))


#Compile
model.compile(loss='categorical_crossentropy', optimizer='Adam',
metrics=['accuracy'])

#fit
filepath='D://JI//newxrd//xrd_model//230labelmodel.out'
modelCheckpoint=keras.callbacks.ModelCheckpoint(filepath,
monitor='val_loss', verbose=0, save_best_only=True, mode='auto')
history = keras.callbacks.History()
model.fit(train_X, train_Y230, batch_size=1000, nb_epoch=5000,
validation_split=0.25, callbacks=[modelCheckpoint,history], shuffle=True)

# check the accuracy
a = model.evaluate(train_X, train_Y230)
print(a)
a = model.evaluate(test_X, test_Y230)
print(a)

#save log after training
acc_log = history.history['acc']
val_acc_log = history.history['val_acc']
loss_log = history.history['loss']
val_loss_log = history.history['val_loss']
acc_log = np.array(acc_log)
val_acc_log = np.array(val_acc_log)
loss_log = np.array(loss_log)
val_loss_log = np.array(val_loss_log)
mat = np.vstack((loss_log, acc_log, val_loss_log, val_acc_log))
mat = np.transpose(mat)
dataframe1 = pd.DataFrame(data=mat)
dataframe1.to_csv('save_log.csv', sep=',', header=False,
float_format='%.7f', index=False)
```

The weight values for the three CNN architectures, if needed by the reader, can be provided by email to the corresponding author.
